# Rewilding relationships: Principles for forging relationships in social-ecological systems

**DOI:** 10.1007/s13280-025-02238-1

**Published:** 2025-09-03

**Authors:** Kristy M. Ferraro, Toryn Whitehead

**Affiliations:** 1 Biology Department, Memorial University, 45 Arctic Ave, St. John’s, NL Canada; 2 Yale University School of the Environment, 370 Prospect Street, New Haven, CT USA; 3 Department of Geography, King’s College London, 40 Aldwych, London, WC2B 4BG UK

**Keywords:** Environmental ethics, Human-nature relationships, Restoration, Rewilding, Socio-ecological systems, Species reintroductions

## Abstract

Rewilding deliberately forges new relationships within complex socio-ecological systems. Yet, many rewilding initiatives proceed without fully considering the multitude of relationships at play. In this paper, we advance a framework that reimagines rewilding as a relationship-centered process, emphasizing that success depends on fostering connections from individual to collective levels for humans and non-humans alike. To illustrate this, we focus on species (re)introductions, identifying the various collective and individual relationships that shape rewilding outcomes. We then propose five principles for effectively forging these relationships: (1) reconsider values and perceptions of nature; (2) embrace a collective and individual-oriented approach; (3) place local communities at the heart of rewilding initiatives; (4) cautiously revive lost relationships; and (5) strengthen the connection between science and policy. Our framework demonstrates that identifying and fostering these relationships is not just essential but transformative, paving the way for rewilding practitioners to create ethical, interconnected, and resilient socio-ecological systems.

## Introduction

As ecosystems worldwide face accelerating loss of biodiversity, biomass, and ecosystem functions (Dirzo et al. 2014; Oliver et al. 2015; Almond et al. 2021; Sellars and Franks 2024), conservationists are increasingly embracing rewilding as a bold and hopeful response (Johns 2019; Perino et al. 2019). The term and concept of ‘rewilding’ began to be widely used in conservation discourse in the 1980s and 1990s—primarily as a strategy to establish connected core wilderness areas for large carnivores (Soulé and Noss 1998). Even before that, in South Asian and African contexts, rewilding had been used to describe the rehabilitation and release of wild predators (Hayward et al. 2019). More recently, rewilding has gained increasing popularity, with projects initiated worldwide to promote ecological restoration and conserve biodiversity (Johns 2019). While the concept of rewilding has evolved (Nogués-Bravo et al. 2016), and it remains a plastic term (Jørgensen 2015), the most common goals are to restore ecosystem functions, natural processes, and species interactions, and, separating it from other restoration projects, to reduce human control and pressure (Lorimer et al. 2015; Carver et al. 2021). There is also an increasing focus on the socio-cultural goals of rewilding linked to human-wildlife coexistence and the positive and negative impacts of rewilding on human well-being (Hawkins et al. 2024).

A central facet of rewilding involves the reintroduction of an extirpated non-human animal species or the introduction of non-native wild, domestic, or semi-domesticated non-human animal species [herein referred to as animals] that perform analog ecosystem functions of an extirpated species [hereafter collectively referred to as (re)introductions] (Perino et al. 2019; Carver et al. 2021). Given that the extirpation of animal individuals, populations, or species can happen at various spatial scales, the spatial scales of rewilding efforts also vary—from large-scale national initiatives that (re)introduce wide-ranging species (such as predator rewilding in Yellowstone) to smaller projects aimed at restoring ecosystem functions through the (re)introduction of semi-domesticated grazers (such as at the Knepp Estate in England). Through such (re)introductions, rewilding initiatives aim to increase biodiversity, re-establish trophic dynamics, and restore ecosystem processes such as nutrient cycling and water (Pettorelli and Bullock 2023; Schmitz et al. 2023). Often, these goals are realized (Svenning et al. 2016; Dvorskỳ et al. 2021), and as a conservation strategy, (re)introductions have demonstrated ecological benefits. As such, rewilding efforts frequently emphasize their functional and utilitarian outcomes.

In contrast, despite rewilding’s commitment to transforming human–nature relationships since its inception (Soulé and Noss 1998), there has been a tendency in practice to only superficially engage with communities and a failure to embed social and cultural elements into projects (Drouilly and O’Riain 2021; Ferrer and Pons-Raga 2022; Martin et al. 2023a; Gonzalez et al. 2024). This narrow focus reflects the influence of Western—and later Western environmental—philosophies that have long upheld a divide between humans and nature (Jørgensen 2015; Ward 2019). In recognition of a need to better engage communities and build lasting rewilding programs, Carver et al. (2021) call for a paradigm shift in the relationship between humans and nature.

**Fig. 1:**
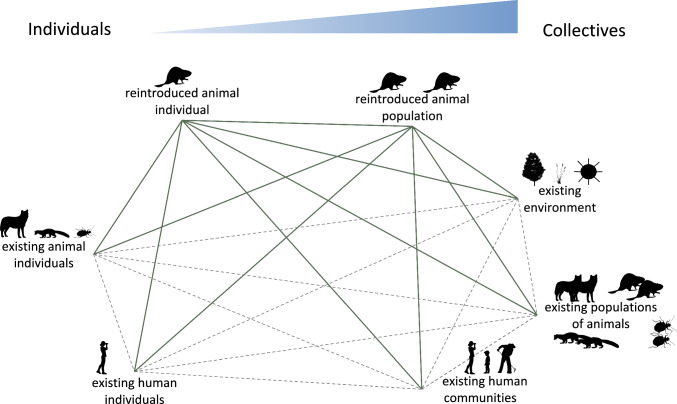
Network of relationships to consider when (re)introducing individual animals or animal populations. Solid green lines represent relationships that are directly forged as a result of the (re)introduction. Dashed gray lines represent relationships that may be indirectly affected by the (re)introduction. We note that there are multiple levels between the individual and the collective level as illustrated by the spectrum. Silhouettes from phylopic.org

To help facilitate this shift, we advance a framework that conceptualizes and evaluates rewilding through the lens of forging new, and revisiting existing, relationships (Ward 2019). At its core, rewilding is a process of building new connections—between (re)introduced animals and their environments, between humans and nature, and between the individual humans and non-humans within social-ecological systems. By focusing on the relational dimensions of rewilding, we draw attention to the distinct connections that are created and reshaped between (re)introduced animals, their environments, and human communities (Fig. 1; Table 1). This perspective moves beyond viewing animals solely as ecological agents and instead aligns with work that emphasizes their roles as active participants in social-ecological systems (Moyano-Fernández 2022; Edelblutte et al. 2023), that considers animal and human welfare alongside their ecological roles (Ferraro 2025; Kiley-Worthington 2025), and that has suggested viewing rewilding from a relational framing (De Cózar-Escalante 2019; Ward 2019). This framing is also aligned with emerging work on more-than-human geographies, as it conceptualizes rewilded landscapes as spaces where humans and non-humans are entangled through individual and collective relationships (Isaacs 2020; Fry 2023). Through this relational lens, rewilding becomes not only a tool for ecological restoration but also a process of cultivating meaningful, reciprocal, and adaptive relationships among humans, animals, and ecosystems (Ward 2019).

**Table 1:** Description of the various relationships forged and altered due to animal (re)introduction

Relationship	Description
Reintroduced Animal(s)—Environment	Animal (re)introductions create new relationships both between the (re)introduced population and the existing ecosystem, and between individual animals and their surroundings. Because animals both shape and are shaped by their environment, failing to account for these interactions can lead to unsuccessful (re)introductions
Reintroduced Animal(s)—Existing Animal(s)	Rewilding creates a multitude of new relationships between the (re)introduced animals and existing animals at the individual, population, and community levels. Each of these new animal relationships and any changes to existing animal relationships must be considered to ensure the desired individual, community, and ecosystem benefits are gained
Reintroduced Animal(s)—Human(s)	Animal (re)introductions produce dynamic and often contested relationships between humans and animals. Successful outcomes depend on inclusive, relationship-centered approaches that work in meaningful partnerships with local communities to co-produce visions for coexistence that allow both people and (re)introduced individuals and collectives to thrive
Human(s)—Environment	Rewilding reshapes human–environment relationships by socially, economically, and culturally reconfiguring landscapes. When carried out in silos, animal (re)introductions can provoke resistance and erode local connections to place, whereas inclusive, place-based approaches have the potential to sensitively enrich existing connections and forge new human–environment relationships
Human(s)—Human(s)	Rewilding could intensify social conflicts, especially when local communities are superficially engaged or excluded from decision-making. Animal (re)introductions that actively seek to foster relationships between different individuals and communities can more openly consider potential tradeoffs and opportunities while building dialog and trust which are crucial for meaningful human-wildlife coexistence

We begin by identifying various collective and individual relationships that require attention. We demonstrate how these relationships shape rewilding outcomes and how the applicability of a relational framing at both the individual and collective levels can create effective rewilding programs. We focus on rewilding efforts that involve the (re)introduction of animal individuals and populations, as this process directly introduces new agents into the ecosystem, reshaping existing ecological and social relationships while forging new ones. However, we acknowledge that similar principles of relationship building could apply to the (re)introduction of plants or alterations that lead to entire ecosystem community shifts via rewilding. Similarly, we do not talk about every individual or collective relationship formed or changed within a (re)introduction, but rather highlight several pertinent relationships to demonstrate the power of viewing rewilding through this relational lens. Of note, many of the practical suggestions we offer for implementing rewilding through a relational lens are already present in existing rewilding efforts. We encourage their broader adoption and a deeper recognition of these practices as inherently relational.

To achieve and execute a rewilding approach that centers relationship building we propose five principles: (1) reconsider values and perceptions of nature; (2) embrace a collective and individual-oriented approach; (3) place local communities at the heart of rewilding initiatives; (4) cautiously revive lost relationships; and (5) strengthen the connection between science and policy. These principles explicitly build on those put forth by Carver et al. (2021) and, as such, also guide the paradigm shift in the relationships between humans, non-humans, and ecosystems needed for successful rewilding.

## New collective and individual relationships

### New relationships between (re)introduced animals and the environment

Rewilding efforts are often centered on the ecological impacts of (re)introduced populations, focusing on their role as collective agents of change in restoring or enhancing ecosystem functions. For instance, rewilding initiatives have introduced beavers (*Castor canadensis*) with the explicit intention of modifying the structure and flow of water systems, thereby creating habitats that support biodiversity (Stringer and Gaywood 2016). While the health of ecosystems and (re)introduced populations are not necessarily in conflict, the emphasis of rewilding tends to fall on ecological outcomes rather than on the well-being of populations or the welfare of the individual animals (Kiley-Worthington 2025). In this way, rewilding can easily fall into the trap of implicitly, and even explicitly, prioritizing the health of ecosystems over animal wellbeing. However, the relationship between animals and ecosystems is not unidirectional. Animals both shape and depend on the abiotic and plant communities in their environments, influencing and relying on factors like water, nutrients, and shelter (Pringle et al. 2023). Without these critical resources from the environment, (re)introduced populations may fail to establish, jeopardizing their survival and the intended ecological benefits. Thus, rewilding efforts that overlook the relationships between animal individuals and collectives and their environments risk undermining both conservation goals and animal welfare.

At the collective level, the relationship between a (re)introduced population and the existing environment is often unpredictable and context-dependent. While rewilding initiatives may aim to restore specific ecosystem processes, outcomes can deviate from expectations due to the complex, dynamic nature of ecosystems (Burak et al. 2024). For example, recent research suggests that the recovery of Yellowstone’s large carnivore assemblage, including the (re)introduction of wolves (*Canis lupus*), has not restored Yellowstone National Park to its previous ecological state, but rather created an alternative stable state (Hobbs et al. 2024; Ripple et al. 2025). Reconceptualizing rewilding as the forging of a reciprocal relationship at the collective level between (re)introduced populations and their environment offers a more adaptive, holistic framework—one that requires a deliberate effort to create conditions for establishment and persistence while embracing the unpredictability of dynamic, emergent interactions.

At the individual level, which is often overlooked in conservation (Ferraro 2024; Orrick et al. 2024), it is specific individual animals that are (re)introduced and then must forge new relationships with their new environment. Research in conservation science and wildlife biology consistently demonstrates that an individual animal’s traits can profoundly influence population dynamics and ecosystem processes (Wolf and Weissing 2012; Hunter et al. 2022). Therefore, it is likely that an individual’s traits affect the relationships (re)introduced animals form in their new environment.

Among these traits, animal personality has been a focal point, with significant implications for conservation and (re)introductions (Collins et al. 2022; Ferraro et al. 2023; Orrick et al. 2024). For example, in the reintroduction of captive-bred swift foxes (*Vulpes velox*), shyer individuals were found to have higher survival rates (Bremner-Harrison et al. 2004). Similarly, shyer animals may engage in less human-wildlife conflict, while bolder individuals may inhabit human-dominated spaces, leading to increased human-wildlife interaction and potential conflict (Honda et al. 2018; Martínez-Abraín et al. 2022). Alternatively, bolder individuals may travel further distances, leading to more successful dispersal. Personality traits can also shape how animals affect ecosystem functions (Sommer and Schmitz 2020). While conservation has begun to successfully integrate animal personality into species conservation, human-wildlife conflict management, and (re)introductions (Merrick and Koprowski 2017; Honda et al. 2018; Collins et al. 2022), the role of individual animal personality in ecological functions remains underexplored (Collins et al. 2022).

Beyond personality, individual animals also exhibit variation in many traits, including physical characteristics and preferences such as food choices (Stewart 1971) and space use (Wat et al. 2020). These individual differences have long been treated as noise when studying the functional role of a species (Raffard et al. 2017). However, this variation is starting to be recognized as ecologically important—for example, larger individuals within a population can drive higher nutrient fluxes (Norkko et al. 2013), and differences in the amount of food consumed and excreted by individuals can significantly influence ecosystem functioning (Raffard et al. 2017). For example, more exploratory individuals tend to travel farther, affecting seed dispersal patterns and nutrient distribution (Poulsen et al. 2021). By viewing rewilding as a process of forging new relationships—shaped by each individual’s unique traits and roles in the ecosystem—conservation efforts can more effectively account for these differences, both facilitating successful (re)introductions and desired restored ecosystem functions.

### New relationships among (re)introduced and existing animals

The (re)introduction of an extant species into an environment where it has been absent for any period of time, or never existed, requires the new population to establish territories and interact with the biotic community in the ecosystem. This process reshapes existing relationships and creates new ones, both among the broader animal community and between native and newly introduced populations. It also alters the individual relationships within and between these groups, shifting behaviors, roles, and interactions (Fig. 1). All scales must be considered to fully understand and navigate the outcomes of reintroduction.

In many cases, the (re)introduction of a new population can significantly and/or unintentionally alter the existing animal community composition. For example, (re)introducing grazers can not only directly increase mammal diversity but can also increase dung beetle biodiversity (Brompton 2018). Similarly, (re)introducing predators can suppress prey species populations (Dula and Krofel 2020). However, (re)introduced species may also outcompete native species for resources or spread rapidly and threaten local biodiversity. For instance, rewilding grazers in productive systems can decrease plant and insect diversity (Van Klink et al. 2016). To mitigate potential unintended or negative impacts on community dynamics, there must be careful consideration and thorough risk assessments of the relationships between the (re)introduced and existing populations. While such assessments are already a common part of rewilding practice, a relational framework could place greater emphasis on inter-population dynamics and help practitioners consider potential risks and tradeoffs more openly.

The (re)introduction of a new population can also influence the dynamics and behaviors of existing populations of species. For instance, the presence of predators can create a “landscape of fear” (Laundre et al. 2010), leading to cascading effects throughout the community and ecosystem (Gaynor 2019; Monk and Schmitz 2022). A well-known example of this is the (re)introduction of gray wolves to Yellowstone National Park, which led to significant behavioral changes in elk (*Cervus canadensis*) populations and initiated trophic cascades, albeit weaker than initially thought (Creel et al. 2005; Brice 2022). However, not all existing species will be affected by the (re)introduction of a new species, and the nature of the interactions between (re)introduced and native populations can vary across different contexts. Community interactions are complex and influenced by factors such as habitat type, species traits, and existing community dynamics. By carefully considering the newly foraged relationship between a (re)introduced population and existing populations, as well as the biotic community, conservationists can model, anticipate, and manage potential interactions and outcomes.

Unsurprisingly, individual animals vary in how they interact with others of their own species, including differences in roles within social groups (Jolles et al. 2020), and in how they interact with individuals of other species in their communities, including variation in responses to predators or competitors (Castellano et al. 2022; Richmond et al. 2022). These differences have critical implications for rewilding efforts, where (re)introduced animals must form relationships with specific individuals, whether these individuals are co-introduced or already present in the ecosystem. For example, pack animals, such as wolves or wild dogs (*Lycaon pictus*), often possess intricate social structures that influence their behavior and survival (Franks et al. 2020). When (re)introducing such species, the success of the initiative may depend on careful consideration of the individuals introduced or the formation of compatible packs. Similarly, animal groups have cultures (Wooster et al. 2024), with unique individuals occupying different cultural roles that can influence conservation success (Brakes et al. 2019; Goldenberg et al. 2022). For instance, in elephant (*Loxodonta africana*) (re)introductions, individuals with strong social bonds can help integrate translocated herds more smoothly (Goldenberg et al. 2022), while young individuals can facilitate group cohesion between existing and released individuals (Goldenberg et al. 2021).

Likewise, individuals of prey species that have been raised in predator-free environments or in captivity often lack appropriate fear responses to predators (Moseby et al. 2015). These individuals may exhibit risky behaviors, such as foraging in exposed areas or failing to recognize predator cues, which can lead to high mortality rates. Mitigating this issue may require targeted pre-release training to reacquaint prey species with predators, fostering the survival skills necessary for coexistence in the wild.

Animals are also active agents, and often have the ability to influence outcomes through adaptive and context-specific behaviors, which is predicated on their sentience, individuality, lived experiences, cognition, sociality, and cultures (Edelblutte et al. 2023). As our understanding of how animal cognitive experience and cultures impacts ecosystem and community dynamics continues to grow (Wooster et al. 2024), our acknowledgment of the individuality of animals must be incorporated into conservation (Orrick et al. 2024). As such, rewilding must take care to consider the behavioral disposition of individual animals (Gao and Clark 2023) to ensure the new relationships between individuals—both among individuals of the same species and between individuals of different species within the community—may be successful.

### New relationships among (re)introduced animals and humans

The (re)introduction of extirpated or non-native replacement species will forge new relationships between humans and the (re)introduced species. This process presents both opportunities and risks for humans, centered on the concept of coexistence. Interpretations of coexistence vary widely across regions and between different species and social groups, and so in practice a universal definition of coexistence is both undesirable and impossible (Glikman et al. 2021). Broadly, though, coexistence is understood to be a situation where “people and wildlife can both survive and flourish in their shared environments” (Gao and Clark 2023: 2). Importantly, negative interactions still occur in this favorable, dynamic state, but they are proactively and effectively managed. Similarly, the burdens of human-wildlife coexistence are not evenly distributed (e.g., Pettersson et al. 2021). Higher densities of both people and (re)introduced animals can increase the potential for conflict, particularly in smaller or more fragmented landscapes where space is limited. A relationship-oriented framework for rewilding acknowledges this dynamism, incorporating the reality that species (re)introductions will forge new relationships and challenge pre-existing relationships that are bound up in a sense of intergenerational continuity connected to place. Such relationships will necessarily evolve over time, encounter moments of tension, and require compromise, mutual adjustment, and sustained efforts to maintain and nurture coexistence.

The reintroduction of white-tailed eagles (*Haliaeetus albicilla*, hereon sea eagles) to northwest Scotland illustrates the tensions that can occur as relationships change. Between 1975 and 2012, the Scottish government and the Royal Society for the Protection of Birds carried out a series of phased (re)introductions. These efforts unfolded against a socio-political backdrop of a crisis in livestock production characterized by destocking and land abandonment and the diversification of upland rural landscapes from predominantly agrarian systems to include recreation, tourism, and conservation (Burton and Wilson 2006; Thomson et al. 2023). In many ways, sea eagles embodied these broader socio-political shifts and challenged the social order of landscapes and interspecies belonging (Fry 2023). For livestock producers, farming necessitates control over non-human life in order to produce, and the reciprocity expressed in the embodied and emotive acts of controlling foxes, for example, “allow them to be placed within the landscape as legitimately belonging” (Fry 2023: 2508). So, while strict legal protections for (re)introduced sea eagles made sense, these protections also denied the autonomy of farming communities and emphasized that sea eagles did not belong. The protections elicited strong emotive reactions of anger and resentment, underscoring the importance of taking time to consider the complexity and plurality of human-nature relationships. (Glentworth et al. 2024).

Given the importance of relational values of belonging, interventions that solely attempt to distribute the financial burdens of coexistence can fail to resolve human-wildlife conflicts (Dickman et al. 2011; Hamm et al. 2024). The expansion of large carnivores across Europe illustrates this point. Compensation programs designed to mitigate the economic impact of livestock losses have been operating since 1970 with mixed results (Bautista et al. 2019), and at times have even perpetuated negative perceptions of large carnivores (Berger 2006). This is partly because these schemes are time-consuming and overly bureaucratic (Milheiras and Hodge 2011; Theodorakea and Von Essen 2016), but also fundamentally fail to address the fact that attitudes are typically influenced more by intangible costs than economic ones (Kansky and Knight 2014). This has led to a shift toward directing resources toward livestock protection measures (e.g., electric fences, livestock guardian dogs, reinforced human presence) which, in contrast to ex post facto systems, are preventative and proactive (Blanco and Sundseth 2023). Of course, livestock losses still occur, and the economic costs of coexistence can be significant for individual farmers. Thus, when compensation payments are administered easily and quickly, they can help to foster tolerance (Dalmasso et al. 2011). Therefore, both compensation and prevention programs, integrated into local, regional, and national participatory processes that are inherently relationship-centered and consider the social and cultural aspects of coexistence, are best placed to mitigate conflict.

When a more holistic approach is taken, new human-wildlife relationships can be forged which empower local communities, allow non-human entities to thrive, and generate new social and economic opportunities. This is well illustrated by the South of Scotland Golden Eagles Project (SSGEP), which has translocated over 40 individuals since 2018 (Barlow 2022). Recognizing that landowner and land manager interests had not been well integrated into the design, management, and implementation of other similar projects, the SSGEP directly engaged with these communities and hired a dedicated member of staff to build trust and dialog. In addition, the ‘Eagle Schools’ program reached over 3000 children in its first three years, forging new connections between the children and eagles, as well as engaging other members of the local community who participated in the schools’ ‘Eagle Days’ where students shared their learning. Additionally, to facilitate the connection between individual human and eagles, the SSGEP used the practice of identifying and naming the (re)introduced animals. Naming can enhance conservation outcomes by allowing individual humans to form personal relationships with the animals, as they begin to see them not just as abstract wildlife but as identifiable members of their shared environment (Benson 2016; Orrick et al. 2024). Moreover, naming individual animals can evoke empathetic responses, fostering emotional and active engagement that has been shown to enhance conservation outcomes (Benson 2016). While the integrated SSGEP approach has not eliminated the illegal persecution of golden eagles in the region, highlighting the intractable nature of conflicts associated with birds of prey in Scotland (Hodgson et al. 2018; Newton 2021), the project’s inclusive and collaborative approach has galvanized community interest and support for golden eagles and maximized economic opportunities through activities and events such as the annual Eagle Festival in Moffat.

### New relationships among humans and the environment

Rewilding not only involves establishing new relationships between humans and the (re)introduced population but also necessitates re-evaluating the relationship between human communities and the landscapes they live and work within. For example, over the past three decades in the Iberá Wetlands, Argentina, the activities underpinning the human community’s relationships with the landscape have shifted from commercial forestry, illegal hunting, and cattle (*Bos*) ranching to nature-based livelihoods when large swathes of land were procured by the Conservation Land Trust (now operating as Rewilding Argentina). After initial apprehension from stakeholders at local and national levels, the Trust built legitimacy and raised support for the project by taking a place-based approach (Pettersson and De Carvalho 2021; Donadio et al. 2022). This approach integrated elements of local culture and history, employed local people, provided environmental education in schools, and generated major investment in infrastructure estimated at 1.5 billion Pesos (Pettersson and De Carvalho 2021). At the same time, by 2017 the rewilding project had (re)introduced more than 200 animals, including pampas deer (*Ozotoceros bezoarticus*), giant otters (*Pteronura brasiliensis*), and giant anteaters (*Myrmecophaga tridactyla*) (Torres et al. 2018). After extensive stakeholder engagement and surveys which found that 95% of respondents support jaguar (re)introduction, including 65% of cattle ranchers, the first jaguar (*Panthera onca*) was released after a 70-year absence in 2021 (Caruso and Pérez 2013; Donadio et al. 2022). Further releases have since bolstered the population to 12 individuals. These practices have created new social, economic, and cultural opportunities for both locals and visitors, transforming their relationships with the landscape and its wild occupants. However, as the scale and profitability of the region have increased, relationships with local communities have begun to deteriorate in favor of external and political actors (Pettersson and De Carvalho 2021). The value of land has also increased, making it difficult for locals to buy property (Pettersson and De Carvalho 2021). This trend could further marginalize local people who lack resources and exacerbate inequalities, with negative repercussions for a human individual’s sense of place and connection to the landscape.

Many rewilding processes are occurring at a much faster pace than in the Iberá Wetlands, more forcefully challenging people’s relationships with nature and their roles within landscapes characterized by order, control, and a history of cultivation. This reckoning is occurring spontaneously through land abandonment and the natural recolonization of large mammals, such as the wolf in the Netherlands (Drenthen 2015), and through illicit (re)introductions, such as those of wild boar (*Sus scrofa*), beavers, and lynx (*Lynx*) in the UK (O’Mahony 2020; Holmes et al. 2024; Whitehead 2025). This rapid change is highly emotive but discursively framed as necessary owing to multiple ecological crises. Yet, if rewilding is enacted in silos without the consent and meaningful involvement of local communities, ill-consideration of how people’s values and identities are tethered to the landscape will spark social conflicts, harm human relationships, and damage rewilding outcomes—e.g. the continued absence of lync in the UK (Drouilly and O’Riain 2021; Whitehead 2025).

The feral rewilding of wild boar in England also illustrates how species abundance reconfigures social relations (O’Mahony 2020). After being absent for hundreds of years, wild boar were (re)introduced both accidentally from farms and deliberately through illegal releases to the Forest of Dean around the late 1990s. Initially, local residents were supportive of their presence, enjoying the novelty of their new neighbor, which socially and ecologically enriched the landscape. However, as the density and range of the population grew and more people experienced negative impacts (e.g., rooting up gardens), wild boars culturally transformed into burdensome pests who made life more difficult. This case study demonstrates the importance of considering how species (re)introductions challenge the established order of landscapes and people’s place within them, with negative consequences when this is not considered for those human-nature relationships, and so by extension, rewilding outcomes.

### New relationships among human groups and individuals

Rewilding has the potential to reshape existing relationships and forge new relationships between human groups and individuals. It is vital to consider these human–human relationships because conflicts over wildlife can often, at their core, be conflicts between humans about wildlife (Hill 2021; Serenari 2024). This is well illustrated by the unlicensed release of beavers in the River Tay in Scotland. The fact is that these illicit actions produced the largest free-living beaver population in Britain and burst the political logjam, allowing further legal (re)introductions of beavers to take place across the UK (Thomas 2022). In turn, this has sparked new levels of interest in beavers, creating new opportunities for recreational activities such as canoe tours which forge new relationships between people and beavers. However, although the Tay beaver population and range have continued to expand, widespread killing and dam destruction are symptoms of the covert nature of the (re)introduction, which has strained relationships and damaged trust between some interest groups (Campbell-Palmer et al. 2023; Holmes et al. 2024). Negative attitudes and low tolerance toward beavers may be a result of direct impacts such as flooded farmland, but the underlying driving forces are often linked to tensions over social identities and beliefs (Zimmermann et al. 2020). Illicit releases, or even (re)introduction projects which are perceived as exclusive and enforced, can damage human relationships and hinder efforts to foster human-wildlife coexistence. These human relationships are not irreparable though, with the rapid formation of multi-stakeholder groups creating opportunities for groups with opposing views to work together, albeit retroactively, to agree shared goals and develop coexistence strategies (Campbell-Palmer et al. 2023).

Relationship-building beyond the conservation community with organizations with competing objectives and world views can be challenging and pose a barrier to rewilding projects’ goals. One way to overcome this could be to dedicate more time and resources to forging personal one-to-one relationships. This process can help to shed people’s organizational labels, creating new opportunities to build trust and dialog, which could yield meaningful collaborations in the future (Staddon 2021). For example, one Scottish land manager from Eastwood et al.’s (2022: 8) study suggested solving intractable problems by taking one’s labels off and “going somewhere remote to share a bottle of whisky”. While unconventional, this sentiment resonates strongly with broader research in trust, which suggests using alternative engagement strategies to tackle identity-based conflicts (Madden and McQuinn 2014).

It is also important to forge personal relationships within the conservation community that spark hope and action. For example, Cairngorms Connect in Scotland has nurtured human relationships by bringing together neighboring land managers to co-develop a 200-year vision for landscape-scale restoration. This has helped to create a stronger sense of community and given individuals within the partnership a greater sense of empowerment and confidence (Eastwood et al. 2022). In addition, by coming together the partnership has been able to shape public discourse and build acceptance for new land management strategies among individuals and organizations (Eastwood et al. 2022). Thus, forging new human relationships within and beyond the conservation community can help scale-up rewilding projects and develop more just and economically feasible visions for rural landscapes.

### The importance of inter-species relationships

Above, we advance a framework for viewing rewilding through a relational lens, which requires the creation and consideration of interspecies relationships at both the individual and collective level. Here, we expound on the nature of these relationships within a Western, modernist worldview. Historically, the dominant human relationship with nature has been one of separation (Jørgensen 2015; Ward 2019) and dominion—an approach rooted in the belief that the natural world exists primarily for human use and benefit (Manfredo et al. 2016; Castelló and Santiago-Ávila 2022). Even more recent frameworks, such as certain versions of stewardship, often retain elements of this hierarchical view, embedding human authority and teleology at their core (Bennett et al. 2018; Castelló 2022). The relational lens we advance departs fundamentally from these paradigms and instead builds on work that has called for wildness to be viewed as a relational concept (Ward 2019), work advancing more-than-human geographies (Isaacs 2020), and the view of rewilding as a collaborative interspecies process (Drenthen 2015; Burlingame 2025; Ferraro 2025). As such, it does not position humans as managers of ecosystems, but rather as partners—individuals and communities embedded within reciprocal relationships (Merchant 1998; Ward 2019). This perspective is particularly important in the context of rewilding, which often seeks to restore ecosystem functions that human activity has disrupted. A relational approach to rewilding recognizes that these functions emerge from the interactions of diverse organisms, each with their own agency (Edelblutte et al. 2023) and ecosystem role. Thus, while successful rewilding may begin with a dominion-esq framework, in which humans actively (re)introduce animals into an ecosystem, it should progress to a stewardship phase, where ongoing care supports their (re)introduction, and ultimately evolve into a partnership and collaboration (Burlingame 2025)—marked by the return of some degree of animal autonomy (DeSilvey and Bartolini 2019).

Viewing rewilding through a relational lens often requires consideration at the individual level, recognizing the unique roles and experiences of each organism within an ecosystem. Given logistical challenges, this approach is more readily applied to certain species and individuals than others. For instance, low-density, solitary animals such as lynx may be more easily conceptualized and managed at the individual level, while abundant species like wild boar, deer, or insects may present challenges to individualized consideration. Further, in the case of rewilding species like insects, individual-level tracking and understanding may be beyond current technological or logistical capacities. While it may not yet be feasible to account for every animal individual, ecologists and conservationists should still strive to consider their collective-level relationships and remain open to advancements in both technology and philosophy that may one day allow for a more nuanced, individual-centered approach.

## Principles for successfully forging new relationships

Here we propose five principles to achieve and execute a rewilding approach that centers relationship building. These principles build on—and are consistent with—the ten principles for rewilding proposed by Carver et al. (2021). While the principles put forth by Carver et al. emphasize key ecological considerations such as restoring ecological processes and anticipating the effects of climate change, they also call for a stronger socio-ecological foundation. This includes fostering local engagement and support, recognizing the intrinsic value of all species and ecosystems, and promoting a paradigm shift in how humans coexist with nature. Our relationship-centered framework for rewilding directly responds to this call. It aims to advance the envisioned paradigm shift and, through the principles outlined below, further articulates how to cultivate the local engagement and support that Carver et al. identify as essential.

The below principles are grounded in Western, English-speaking epistemologies and are supported by case studies primarily from the United Kingdom and the USA, where we live and work. While we have engaged with epistemologies beyond our own, we want to be clear that these principles may not be universal or comprehensive. That being said, we believe the concept of relationships is broadly applicable, and thus, our framework of viewing rewilding through a relational lens holds relevance beyond our own epistemologies and empirical cases.

### Principle 1: Reconsider values and perceptions of nature

Conservation efforts, including rewilding, are deeply rooted in societal, cultural, and ethical values that shape how humans perceive and interact with the natural world (Noss 2007; Carver et al. 2021; Ferraro et al. 2023). These values influence whether our relationships with ecosystems and animals are characterized by dominion, stewardship, or partnership (Manfredo et al. 2016; Bennett et al. 2018; Castelló and Santiago-Ávila 2023). To foster the new relationships needed for rewilding to succeed, practitioners must reconsider some of the values underpinning conservation. Specifically, conservationists and rewilding practitioners must embrace ecological dynamism over stability, recognize the intrinsic value of individual animals, and move beyond anthropocentric worldviews.

Historically, conservation has focused on maintaining or restoring ecosystems to a perceived ideal, static state (Leopold 1949). This perspective shaped early rewilding practices, which prioritized restoring ecosystem integrity, understood to be a stable ecological state (Carver et al. 2021). However, ecosystems are inherently dynamic, continually evolving in response to environmental changes and community interactions. As such, contemporary conservation ethics challenges both the concept and practicality of pursuing ecological integrity (Carver et al. 2021; Rohwer and Marris 2021). Instead, there is growing acceptance of novel ecosystems—emergent systems that reflect nature’s dynamism and resilience (Hobbs et al. 2009). Rewilding already incorporates this idea to some extent, as seen in the (re)introduction of non-native species to fulfill ecological roles (Schlaepfer et al. 2011). This embrace is particularly emphasized by Carver et al. 2021, who call for the explicit incorporation of ecosystem dynamism in rewilding. At the same time, rewilding often draws inspiration from, and sets benchmarks based on, past ecosystems (Lorimer et al. 2015), revealing a tension between the creation of novel ecosystems and static assumptions about what nature should look like.

Further, because rewilding operates within the broader umbrella of conservation, the success of rewilding initiatives in embracing ecological dynamism depends not only on the intentions of individual practitioners but also on the values, policies, and institutional legacies of the field. Thus, for rewilding initiatives to fully realize their potential, the wider field of conservation must undergo a deeper value shift. This includes moving away from valuing and pursuing an idealized, historical ecosystem and instead embracing uncertainty and ongoing change as core features of ecosystems. It requires letting go of static baselines and fostering adaptive relationships that are grounded not in control, but in curiosity and flexibility.

At the same time, there is a growing consensus within conservation ethics and environment philosophy regarding the moral status of wild animals (Ramp and Bekoff 2015; Wallach et al. 2018; Ferraro et al. 2023). Rather than viewing animals as mere tools to achieve ecosystem functions, this perspective recognizes an animal’s inherent worth *and* contributions to ecological processes (Wallach et al. 2018; Ferraro et al. 2023; Ferraro 2025). We argue this perspective should be integrated into rewilding. While some may view rewilding as a conservation tool that treats animals as a means to an end (e.g., using animals to restore ecosystem function), recognizing the intrinsic and instrumental value of animals is not necessarily at odds. Rather, rewilding projects are likely to be most successful when respecting and valuing the contributions of individual animals to the ecosystem as this approach encourages a deeper consideration of the needs and trade-offs that inevitably arise in rewilding projects, leading to more thoughtful and ethical implementation.

We do not wish to ignore the fact that sacrifices may still need to be made by individuals in rewilding—including the (re)introduced animals, the existing animals, and humans. Trade-offs are an unavoidable aspect of rewilding. For example, the (re)introduction of large carnivores may provide ecosystem functioning by restoring trophic cascades, but also increase stress and mortality risk for naive prey species, disrupt the behavior of existing animal communities, and create instances of human-wildlife conflict. Finding solutions that navigate and reconcile the intrinsic value of individuals and the practical need for sacrifices is challenging. However, in circumstances where there are competing values wildlife management must engage with, rather than retreat from, these tensions. Work on value pluralism in environmental ethics has begun to pave a path for such engagement (Norton 2005; Robinson 2011). Further, work within conservation ethics has begun to demonstrate how recognizing the intrinsic value of non-human individuals can be incorporated in conservation practice (Wallach et al. 2018; Ferraro 2024; Orrick et al. 2024), including the development and advancement of applied compassionate conservation (Wallach et al. 2018; Wallach et al. 2018; Bekoff 2019; Rohwer and Marris 2019). Regardless, we wish to highlight that by acknowledging the intrinsic value of all individuals involved in rewilding, and by considering the relationships between all individuals and collectivities, conservationists are more likely to anticipate and address unnecessary potential harms in the planning process. This might include strategies such as soft-release protocols, ensuring refuge habitats exist for prey, or more gradual introductions to minimize suffering and shock.

It is also necessary to confront the anthropocentric biases that shape conservation norms (Washington et al. 2021; Ferraro et al. 2023). Humans tend to more readily form relationships with large, charismatic megafauna, making it easier to support or reject their reintegration into landscapes. However, every species plays a role in ecosystem functioning, regardless of whether it is perceived as charismatic. For rewilding efforts to be ethically and ecologically grounded, policymakers and practitioners must cultivate a broader appreciation for the intrinsic value and functional roles of all species.

### Principle 2: Embrace a collective and individual-oriented approach

Conservation has traditionally prioritized ecological collectives over individuals, focusing on populations, species, and ecosystems rather than the unique traits of individual animals (Wallach et al. 2018; Ferraro et al. 2023). However, integrating collectivist and individual-oriented perspectives in rewilding can increase success compared to traditional approaches (Pooley et al. 2021; Ferraro 2024; Orrick et al. 2024). An individual-oriented perspective acknowledges that individual humans and animals have distinct personalities, behaviors, and responses to environmental variables. In rewilding practice, taking an individual-oriented approach might involve evaluating whether certain behavioral traits make an animal more or less likely to succeed after (re)introduction or anticipating how its (re)introduction could alter relationships within an ecosystem. For instance, relocating a bold predator from an area of human-wildlife conflict to a rewilding site may appear to be a win–win: resolving conflict in the original landscape while contributing to restoration elsewhere. However, boldness may also increase the likelihood of new conflicts in the release site, depending on human tolerance. Evaluating such traits is difficult, and the tools for measuring animal personality are still developing. Nonetheless, when a particular trait is possibly linked to the success or failure of a rewilding imitative, it is worth considering in order to improve outcomes, minimize unintended consequences, and safeguard the welfare of both the animals and people involved.

A renewed focus on individuals could also be fruitful for the human dimensions of rewilding because human experiences and impacts are deeply personal and localized (Orrick et al. 2024). For example, rewilding projects that seek to translocate or (re)introduce predators, such as sea eagles or lynx, can place additional pressures on rural livelihoods and challenge people’s sense of place in the landscape (Bavin et al. 2023; Fry 2023). However, it is not farming communities as a whole that bear the brunt of predation, but individual farmers who face the financial and emotional burdens of losing animals to predators. Taking an individual-focused approach allows for more tailored solutions that address specific concerns (see Orrick et al. 2024). This personalized approach also builds trust, making individuals feel that their unique challenges are being understood and addressed. By taking time to forge personal relationships, rewilding efforts can become more collaborative endeavors that effectively address the desires and concerns of individuals living in the landscape.

By addressing both the collective needs of species and the distinct characteristics of individual humans and animals, rewilding efforts can be more precisely tailored to ensure successful establishment, foster positive human-wildlife interactions (Orrick et al. 2024), and support the creation of resilient, self-sustaining ecosystems. This holistic approach ensures that the nuances of individual behavior are not overlooked, leading to more effective and adaptable conservation strategies.

### Principle 3: Place local communities at the heart of rewilding

Placing local communities—both human and animal—at the heart of rewilding efforts fosters relationships that are crucial for the success and sustainability of these initiatives. To do this, rewilding must look beyond superficial notions of inclusivity that treat human community engagement as a tick-box exercise and instead build collaborative partnerships with communities (Hawkins et al. 2024). This is especially important given rewilding’s reputation among certain groups as elitist (Wynne-Jones et al. 2018; Holmes et al. 2020), with aspirations of meaningful collaboration often limited by entrenched views of power, land ownership, and self-interest (Martin et al. 2023a). When rewilding projects enact place-based approaches, they can harness Indigenous and local knowledge and practices to improve the quality and inclusivity of interventions (Hawkins et al. 2024). For example, the (re)introduction of bison (*Bison Bison*) to Banff National Park in Canada more than 130 years after their extirpation was guided by an Indigenous Advisory Circle. Activities such as blessing ceremonies integrated knowledge and beliefs from historical experiences of coexistence into the initiative (Heuer et al. 2023). The restoration of the relationship between humans and bison has helped to create a more resilient and diverse foundation for the rewilding initiative, demonstrating that when Indigenous communities are central to rewilding efforts, it creates a synergistic relationship where both nature and human communities thrive.

However, while Indigenous communities’ knowledge and rights are increasingly recognized within conservation policy and practice, local communities whose cultures, identities, and livelihoods are distinct and intimately tied to nature continue to lack commensurate recognition (Pettersson et al. 2025). This fuzzy understanding of ‘who is local’ has often resulted in the exclusion of local knowledge holders from decision-making processes, whose knowledge is disregarded as anecdotal and contradictory to the mainstream conservation agenda (Martin et al. 2023b; Pettersson et al. 2025). Underpinning the exclusion—or superficial inclusion—of local knowledge within rewilding practice has been a tension between competing visions for the future of rural landscapes underpinned by “different interpretations of the environment as a horizon for self-understanding” (Drenthen 2015: 412).

In the Cambrian mountains of west Wales, there are two distinct visions for the area (Holmes et al. 2022). The ‘socio-ecological rebalancing’ vision is opposed to perceived industrial monocultures, including sheep grazing, which is seen as preventing the transformation of rural landscapes to nature-rich ones populated by locally extinct species and characterized by the expansion of oak woodlands. The ‘managing farming heritage landscapes’ vision is strongly in favor of traditional farming and its heritage, which over multiple-generations of human involvement has created meaningful places deeply intertwined with people’s sense of place and identity. When the Summit2Sea (S2S) landscape restoration project was first launched in 2018, it faced stark opposition from local communities which led to several partners withdrawing, including Rewilding Britain, and the subsequent reframing of all its ideas and dropped all mentions of rewilding. Principally, opposition to the project was rooted in its underappreciation of situated knowledge (Jones 2022), as well as its potential disruption of the relationships that existed locally between human and non-human (principally sheep, *Ovis aries*) individuals and communities, and the landscape—the perceived removal of human traces and creation of “meaningless landscapes” (Drenthen 2015: 412).

By conceptualizing rewilding as a relational process, it becomes easier to identify these unavoidable and recurrent points of tension and recognize that dialogue and tradeoffs are an inherent part of the process; a focus on relationships encourages a more adaptive and collaborative mindset. One tool that could prove invaluable is knowledge braiding, which moves beyond integration to value the complementarity of different knowledge systems (Pettersson et al. 2025). To do this, equitable partnerships anchored in place and local relations should be organized through ‘communities of practice’ where emplaced practitioners facilitate a process in which to “jointly formulate questions, select methods, collect and analyze data, as well as share, reflect and learn from each other” to weave local and scientific knowledge (Pettersson et al. 2025: 6). This process should include the co-creation of more holistic measures of success which include the affective and material aspects of social-ecological transformations. The S2S project illustrates this process well. After the withdrawal of Rewilding Britain, a facilitated co-design process which centered relationship and trust building produced a shared vision for the landscape rooted in the local place and culture of west Wales (Tir Canol 2021). This transformation is embodied by the renaming of the project by the local community to Tir Canol—translated as ‘land’ ‘middle’—reflecting the middle ground achieved through the revived project’s collaborative and inclusive approach.

### Principle 4: Cautiously foster new relationships

In order to yield the ecological and socio-ecological benefits of (re)introduced species, policymakers and rewilding practitioners must cautiously foster new relationships. Yet, in many areas where extirpated species could be (re)introduced, people no longer have any knowledge, memories, experiences, or connections to these species. This extinction of experience could have significant implications for human-wildlife coexistence (Whitehead and Hare 2025). For example, in areas of Spain where wolves have been extirpated, the loss of traditional ecological knowledge has led to less tolerance of wolves and awareness of the benefits they provide (Durá-Alemañ et al. 2024), whereas in areas where wolf presence is uninterrupted, traditional ecological knowledge was important for reducing conflict and fostering a more favorable state of coexistence (Durá-Alemañ et al. 2024).

This more favorable state of coexistence should not occlude the fact that negative attitudes may be prevalent and that human-wildlife conflict, including legal and illegal killing, still occurs in areas of sustained human-wildlife coexistence. People living in areas where wolves have never been extinct have more negative attitudes than people living in areas where there are no wolves, or where wolves have recolonized (Barmoen et al. 2024). Nevertheless, emerging research appears to indicate that there are important lessons that can be learned from areas with sustained human-wildlife coexistence—such as livestock farmer’s tolerance to livestock depredation events (Torres et al. 2018; Durá-Alemañ et al. 2024; Pettersson et al. 2025).

The extinction of experience also extends to missing social and cultural features of landscapes which are vital for coexistence (Whitehead and Hare 2025). In Scotland, for example, livestock grazing systems have developed for over 400 years in the absence of large carnivores, resulting in larger herds and an absence of protection measures. From this perspective, the (re)introduction of large carnivores to Scotland poses significant challenges. Any (re)introduction would place additional financial and emotional burdens on farmers who would need to learn, practically and culturally, to live with large carnivores. One action that may aid this process could be fieldtrips where farmers from an area where a (re)introduction is proposed visit farmers in a different country where they currently, and historically, coexist with large carnivores. This peer-to-peer knowledge exchange could help to inform farmers from the proposed reintroduction site, dispelling some concerns as well as potentially raising new ones. However, disseminating the knowledge and experiences gleaned by the few farmers who attend these fieldtrips to the wider local community remains challenging, although, audio-visual technologies, such as videos of European farmers discussing human-lynx coexistence used in *The Missing Lynx Project’s* traveling exhibition, may help to do this.

To overcome these barriers, rewilding projects should first and foremost focus on building transparent and trusting relationships with land managers and other interested groups. Forging these personal relationships is a critical foundation for a collaborative process that informs all stakeholders about the extirpated animal, its behavior, and what coexistence could look like, as well as co-producing strategies to minimize conflict and fairly distribute the burdens of coexistence—aided by the provision of financial and psychological support. Any attempt to bypass this longer process through illegal releases risks sparking human-wildlife conflicts that otherwise may have been avoided or mitigated (e.g., Holmes et al. 2024). Inevitably, illegal releases, whether they are successful or not, can strain human relationships and undermine evidence-based conservation more generally (Sutherland et al. 2025; Whitehead 2025). By enacting impartial, long-term processes of facilitated dialog that build trust, preparedness, and consensus, rewilding projects can create more solid foundations from which to foster new human-wildlife relationships.

### Principle 5: Strengthen the connection between science and policy

Policy is crucial for conservation because it establishes the legal framework and guidelines for protecting ecosystems, wildlife, and natural resources. The policy landscape also shapes human and non-human relationships, and if the current mix of policy instruments is not cohesive, or is even counterproductive, it could be perpetuating existing human-wildlife conflicts (IUCN 2023). Therefore, for rewilding to achieve a paradigm shift in the relationship between humans and nature, there must be a stronger connection between science–including traditional ecological knowledge and local knowledge–and policy. This poses a number of challenges since rewilding itself is considered a ‘toxic’ term by some, and there is currently a lack of empirical evidence informing rewilding theory and practice (Pettorelli et al. 2018; Sandom et al. 2019). Furthermore, within conservation itself the relationship between evidence and practice is complex and messy, imbued in values, identities, power structures, and cultural differences (Adams and Sandbrook 2013; Kyle and Landon 2023). This, combined with a culture of policy-led science where decision-makers cherry-pick whatever version of science best fits at that moment in time (Boyd 2024), has at least in part led to the poor articulation of rewilding in many national policies, such as in the UK ((Cary and Wartmann 2024).

To facilitate wider-spread implementation of rewilding, it may be fruitful to evaluate policies relating to the key components of rewilding, such as native species restoration in the Endangered Species Act in the USA and landscape restoration in the 30 × 30 target in the Global Biodiversity Framework (Carlson et al. 2023; Bachmann-Vargas et al. 2024). It is critical to keep relationships at the forefront of this policy development process. Although this endeavor is aided by the adoption of relationality within the conceptual framework of the IPBES, one way to achieve relationship-centered policy development and strengthen the connection between science and policy could be to make the process more inclusive. This can be achieved by adopting a broader definition of evidence to include historical documents and artifacts, traditional environmental knowledge, and local knowledge (Pauly 1995; Adams and Sandbrook 2013; Carver et al. 2021). In addition, deliberative democracy, such as people’s assemblies, can embed procedural and recognitional justice into decision-making by increasing participation, especially of marginalized voices, and increasing the diversity of experiences and knowledge involved in the process (Ross et al. 2021). Non-human actors are often poorly represented in these policy-making processes, so it is also important to consider whose knowledge of non-human others is regarded as legitimate and who is authorized to speak on their behalf (Staddon 2021).

## Conclusion

Animal (re)introductions have the potential to do some of the hard work and heavy lifting to reverse biodiversity decline and ecosystem function loss (Perino et al. 2019; Egoh et al. 2021; Schmitz et al. 2023). They can also enrich landscapes for people, offering new wildlife experiences and socioeconomic opportunities (Pettersson and De Carvalho 2021; Barlow 2022; Eastwood et al. 2022). Yet conservation is reckoning with the need to repair damaged relationships—both between humans and natural entities, and natural entities themselves that have been harmed by humans. Thus, rewilding is more than just an ecological intervention—it is a process of mending and re-establishing connections. By adopting a relationship-focused lens, rewilding initiatives can acknowledge the interconnectedness of individuals and collectives, enabling more nuanced and successful approaches that restore the biological and socio-ecological complexity of ecosystems.
